# A Role for the Unfolded Protein Response (UPR) in Virulence and Antifungal Susceptibility in *Aspergillus fumigatus*

**DOI:** 10.1371/journal.ppat.1000258

**Published:** 2009-01-09

**Authors:** Daryl L. Richie, Lukas Hartl, Vishukumar Aimanianda, Michael S. Winters, Kevin K. Fuller, Michael D. Miley, Stephanie White, Jason W. McCarthy, Jean-Paul Latgé, Marta Feldmesser, Judith C. Rhodes, David S. Askew

**Affiliations:** 1 Department of Pathology & Laboratory Medicine, University of Cincinnati College of Medicine, Cincinnati, Ohio, United States of America; 2 Unité des Aspergillus, Institut Pasteur, Paris, France; 3 Division of Infectious Diseases, Department of Medicine, University of Cincinnati College of Medicine, Cincinnati, Ohio, United States of America; 4 Division of Infectious Diseases, Department of Medicine, Albert Einstein College of Medicine, Bronx, New York, United States of America; 5 Department of Microbiology and Immunology, Albert Einstein College of Medicine, Bronx, New York, United States of America; 6 Department of Obstetrics & Gynecology and Women’s Health, Albert Einstein College of Medicine, Bronx, New York, United States of America; David Geffen School of Medicine at University of California Los Angeles, United States of America

## Abstract

Filamentous fungi rely heavily on the secretory pathway, both for the delivery of cell wall components to the hyphal tip and the production and secretion of extracellular hydrolytic enzymes needed to support growth on polymeric substrates. Increased demand on the secretory system exerts stress on the endoplasmic reticulum (ER), which is countered by the activation of a coordinated stress response pathway termed the unfolded protein response (UPR). To determine the contribution of the UPR to the growth and virulence of the filamentous fungal pathogen *Aspergillus fumigatus*, we disrupted the *hacA* gene, encoding the major transcriptional regulator of the UPR. The *ΔhacA* mutant was unable to activate the UPR in response to ER stress and was hypersensitive to agents that disrupt ER homeostasis or the cell wall. Failure to induce the UPR did not affect radial growth on rich medium at 37°C, but cell wall integrity was disrupted at 45°C, resulting in a dramatic loss in viability. The *ΔhacA* mutant displayed a reduced capacity for protease secretion and was growth-impaired when challenged to assimilate nutrients from complex substrates. In addition, the *ΔhacA* mutant exhibited increased susceptibility to current antifungal agents that disrupt the membrane or cell wall and had attenuated virulence in multiple mouse models of invasive aspergillosis. These results demonstrate the importance of ER homeostasis to the growth and virulence of *A. fumigatus* and suggest that targeting the UPR, either alone or in combination with other antifungal drugs, would be an effective antifungal strategy.

## Author Summary

The pathogenic mold *Aspergillus fumigatus* is the leading cause of airborne fungal infections in immunocompromised patients. The fungus normally resides in compost, an environment that challenges the organism to obtain nutrients by degrading complex organic polymers. This is accomplished by secreted enzymes, some of which may also contribute to nutrient acquisition during infection. Extracellular enzymes are folded in the endoplasmic reticulum (ER) prior to secretion. If the folding capacity of the ER is overwhelmed by increased secretory demand, the resulting ER stress triggers an adaptive response termed the unfolded protein response (UPR). In this study, we uncover a previously unknown function for the master transcriptional regulator of the UPR, HacA, in fungal virulence. In the absence of HacA, *A. fumigatus* was unable to secrete high levels of proteins and had reduced virulence in mice. In addition, loss of HacA caused a cell wall defect and increased susceptibility to two major classes of antifungal drugs used for the treatment of aspergillosis. These findings demonstrate that *A. fumigatus* relies on HacA for growth in the host environment and suggest that therapeutic targeting of the UPR could have merit against *A. fumigatus*, as well as other eukaryotic pathogens with highly developed secretory systems.

## Introduction

*Aspergillus fumigatus* is a soil-dwelling filamentous fungus that has become the predominant mold pathogen of the immunocompromised population [1],[2],[3]. The infection is acquired through the inhalation of aerosolized conidia (spores), which are small enough to reach the distal airways [4]. When the inhaled conidia germinate and develop into hyphae, secreted fungal hydrolases progressively damage the integrity of the pulmonary epithelium, allowing vascular invasion with subsequent hematogenous spread [4],[5]. Despite the introduction and use of recently approved antifungals, invasive aspergillosis (IA) continues to be associated with a poor outcome [1],[3],[6],[7]. Moreover, the incidence of IA is expected to rise with the expansion of the immunosuppressed population, making the search for novel treatments a high priority. Unfortunately, few effective drugs are identifiable in the late-stage development pipeline [8], emphasizing the need for increased understanding of the virulence of this organism to facilitate the rational design of novel therapeutic strategies.

The prevailing evidence suggests that the virulence of *A. fumigatus* involves gene products that have evolved to enhance the competitiveness of the fungus in the ecologically diverse environmental niche of decaying organic debris. The saprophytic nature of this lifestyle requires the secretion of abundant enzymes that enable the fungus to extract nutrients from complex polymeric material [5],[9],[10]. This high capacity secretory system has been exploited in other filamentous fungi for the industrial production of native and heterologous proteins and is a feature that distinguishes these organisms from the yeast *Saccharomyces cerevisiae* [11],[12],[13], in which secretion levels are sometimes too low for industrial application [14]. As in all eukaryotes, the endoplasmic reticulum (ER) of filamentous fungi is the major processing center for secreted and transmembrane proteins. The unique environment of the ER facilitates the folding of nascent proteins, a process that is aided by ER-resident chaperones and folding enzymes, and post-translational modifications such as glycosylation, phosphorylation and disulfide bridge formation [15]. From the ER, proteins are transferred to the Golgi compartment, where they undergo further processing before being delivered to the membrane by vesicles of the distal secretory system. Under normal conditions, the secretory demand is balanced by the protein folding capacity of the ER. However, as much as a third of newly synthesized proteins fail to achieve native structure due to imperfections in transcription, translation, post-translational modifications or protein folding [16]. Thus, an inevitable consequence of a high rate of protein synthesis, coupled with a rapid flux through the secretory system, is the accumulation of misfolded proteins in the ER. Unfolded proteins threaten cell survival because they form toxic aggregates that interfere with the function of normal proteins [17]. If the influx of nascent unfolded polypeptides exceeds the folding capacity of the ER, the ensuing ER stress triggers a series of adaptive responses collectively termed the unfolded protein response (UPR) [18].

The UPR is a conserved eukaryotic signaling pathway that originates in the ER and transmits information on the folding capacity of the secretory system to the nucleus. Upon activation, the UPR restores ER homeostasis by reducing the flow of proteins into the ER, increasing protein transport out of the ER, increasing the expression of ER-resident chaperones and foldases, and by degrading proteins that fail to properly fold [18],[19]. The UPR affects the secretory pathway at multiple levels and has been shown to involve at least 381 genes in *S. cerevisiae*, underscoring the complexity and importance of this pathway for cell survival [20]. The upstream ER sensor responsible for detecting unfolded proteins and triggering the UPR is Ire1p, a transmembrane protein that has an ER lumenal sensing domain and a protein kinase and endoribonuclease domain in the cytoplasmic region [21]. Activation of the sensor is triggered by interaction with unfolded proteins in the ER lumen, possibly facilitated by the dissociation of ER-resident chaperones from Ire1p [19]. These events elicit Ire1p aggregation and *trans*-autophosphorylation, resulting in activation of the cytosolic ribonuclease domain [22],[23]. The increased ribonuclease activity catalyzes the spliceosome-independent cleavage of the cytoplasmic precursor mRNA *HAC1u* (uninduced), removing a single intron to generate the induced form of the *HAC1* mRNA, *HAC1i* (induced) [24]. This unconventional splicing event creates a frame-shift in the mRNA, allowing for the translation of a transcription factor that moves to the nucleus and regulates the expression of UPR target genes [20],[25].

Despite advances in our understanding of the nature and scope of the UPR in several organisms, the significance of this pathway to the virulence of a pathogenic eukaryote is unknown. In this study, we generated a mutant of *A. fumigatus* that is deficient in UPR signaling by disrupting the *hacA* gene, encoding the ortholog of the yeast Hac1p transcription factor responsible for modulating the expression of UPR target genes. The results demonstrate that *A. fumigatus* relies heavily on the UPR to sustain growth under conditions that disrupt ER homeostasis, including thermal stress, cell wall stress and a high secretory load. In addition, loss of the UPR was associated with attenuated virulence and a dramatic increase in antifungal drug sensitivity. Taken together, these data provide evidence that *A. fumigatus* is under ER stress *in vivo* and would, therefore, be vulnerable to therapeutic attack on the fungal UPR.

## Results

### Disruption of the UPR by Deletion of *hacA* in *A. fumigatus*

**Figure 1:**
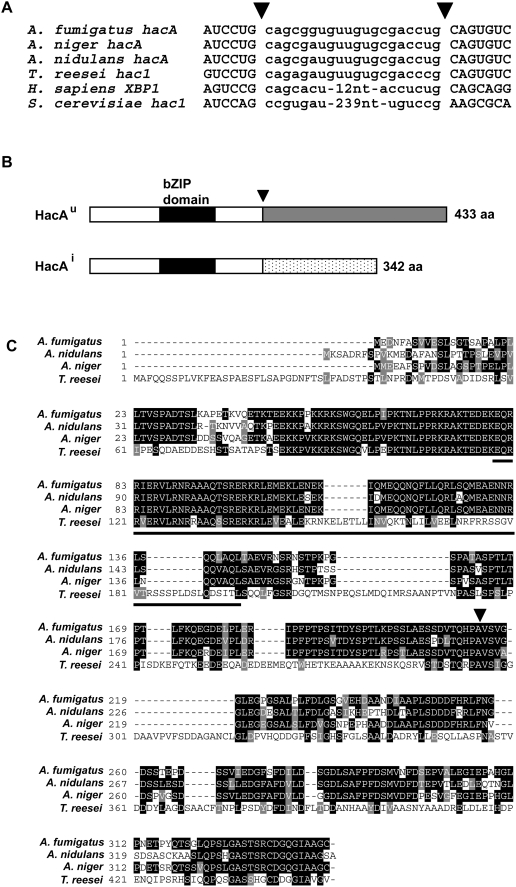
Sequence analysis of *A. fumigatus hacA^i^*. (A) Alignment of the RNA sequence surrounding the unconventional intron in *A. fumigatus*, *A. nidulans*, *A. niger*, *Trichoderma reesei*, *H. sapiens*, and *S. cerevisiae*. Intron sequences are shown in lower case. (B) Schematic representation of the predicted *A. fumigatus* HacA^u^ protein (433 amino acids) and the HacA^i^ protein (342 amino acids). Black box: bZIP domain. Grey box: unique c-terminus of HacA^u^. Stippled box: unique c-terminus of HacA^i^. Downward arrow denotes the location of the unconventional intron. The Genbank accession number for the *A. fumigatus hacA^i^* cDNA sequenced in this study is EU877964. (C) Multiple sequence alignment comparing the HacA protein of *A. fumigatus* with orthologs from *A. nidulans* (AJ413273), *A. niger* (AY303684), and *T. reesei* (AJ413272). Black boxes denote identical amino acids, and grey boxes denote similar amino acids. The sequence was aligned using DNAMAN software (Lynnon Corp, Canada) using default parameters. Output order was based on input and was exported in CLUSTALW format for shading using BOXSHADE 3.21 (http://www.ch.embnet.org/software/BOX_form.html). The underlined sequences represent the predicted bZIP domain and the downward arrow indicates the unconventional splice site.

The UPR-induced (*hacA^i^*) and uninduced (*hacA^u^*) forms of the *A. fumigatus hacA* mRNA were cloned by RT-PCR from RNA derived from cultures grown in the presence or absence of dithiothreitol (DTT)-induced ER stress, respectively. A comparison of the cDNA sequences with the *A. fumigatus* genome revealed a conventional intron with consensus border sequences that is excised in both the *hacA^u^* and *hacA^i^* mRNAs. In addition, an unconventional 20 nt intron is uniquely excised from the *A. fumigatus hacA^i^* mRNA in response to ER stress (Figure 1A), similarly to what has been reported in other orthologs of this mRNA [26]. This atypical intron is much smaller than the corresponding 252 nt intron of *S. cerevisiae HAC1* [24], but is similar in size to introns that are spliced by UPR activation in mammals (26 nt) [27], *Caenorhabditis elegans* (23 nt) [27], *Candida albicans* (19 nt) [28] and filamentous fungi (20 nt) [29],[30]. The exact splicing sites of the unconventional intron in *A. fumigatus* could not be unambiguously identified by comparing cDNA and genomic sequences because of the presence of a CTGCAG at each side of the intron, a feature that is also found in other filamentous fungi [29],[30]. Although the size of the intron varies between genera, the border sequences are highly conserved (Figure 1A) and are located in a region of strong predicted RNA secondary structure (data not shown) [29],[30].

The first 213 amino acids encoded by the *hacA* ^u^ and *hacA* ^i^ mRNAs are identical. This region contains a leucine zipper dimerization motif adjacent to a basic DNA binding domain (Figure 1B), which is characteristic of bZIP-type family transcription factors. The atypical splicing of the *A. fumigatus hacA^i^* mRNA changes the reading frame, resulting in an encoded protein that replaces 220 amino acids at the c-terminus of HacA^u^ with a unique c-terminal domain comprised of 129 amino acids (Figure 1B). The resulting HacA^i^ protein has 76% and 81% identity to the corresponding proteins in *A. nidulans* and *A. niger*, respectively. Alignment of the *A. fumigatus* HacA^i^ protein with orthologs from other filamentous fungi reveals extensive homology throughout the protein (Figure 1C). By contrast, most of the homology to *S. cerevisiae* Hac1p is concentrated in the DNA binding domain (data not shown).

**Figure 2:**
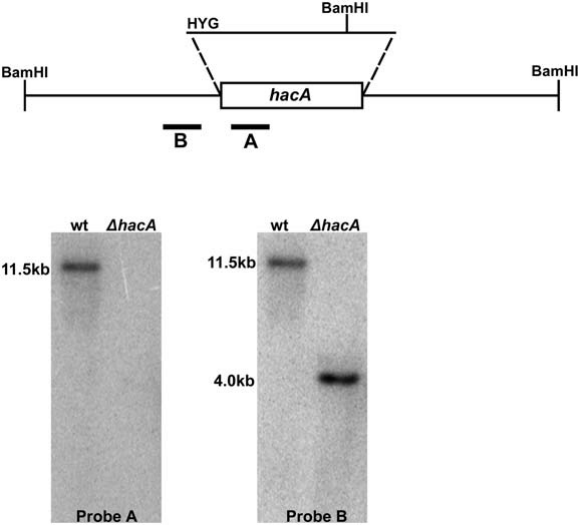
Disruption of *hacA*. Deletion strategy: The *hacA* gene was deleted by replacing the coding region with the hygromycin resistance cassette (HYG). Southern blot analysis of *Bam*HI–digested genomic DNA using Probe B (flanking region) identified the predicted 11.5 kb wt band which was truncated to 4.0 kb in the *ΔhacA* mutant. A second probe (Probe A) derived from the deleted region of the *hacA* gene was used to confirm that no duplication had occurred.

**Figure 3:**
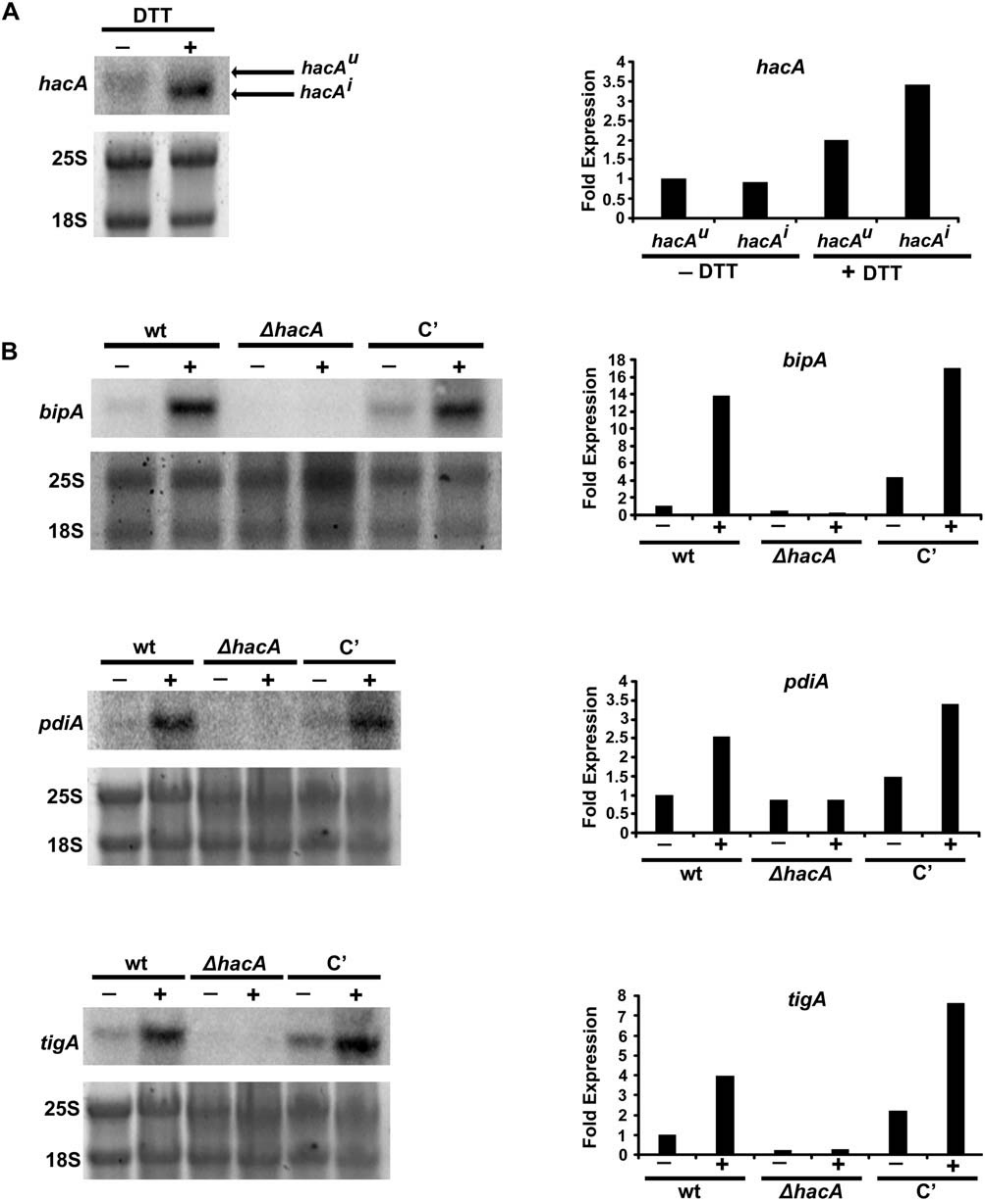
Loss of *hacA* disrupts UPR signaling in *A. fumigatus*. (A) Northern blot analysis of *hacA* expression and processing in the presence (+) or absence (−) of 1mM DTT for 1 h. Hybridization intensity was determined by phosphorimager analysis, and is presented graphically as fold expression relative to the levels of *hacA^u^* in the absence of DTT. (B) Northern blot analysis of UPR target gene expression (*bipA*, *pdiA*, and *tigA*) in the presence (+) or absence (−) of DTT. Hybridization intensity is presented graphically as fold expression relative to the wt strain in the absence of DTT.

Deletion of *hacA* was accomplished by replacing the *hacA* ^i^ open reading frame with the hygromycin resistance cassette (Figure 2). To determine whether loss of *hacA* was sufficient to disrupt UPR signaling in *A. fumigatus*, the expression of four known UPR target genes was examined by northern blot analysis, including *bipA* (ER chaperone), *pdiA* and *tigA* (protein disulfide isomerases) and *hacA* itself. Each of these genes contains an unfolded protein response element (UPRE) in its promoter [31], and the abundance of each mRNA increases in response to UPR activation [30]. As expected, treatment of wt *A. fumigatus* with DTT increased *hacA* abundance and induced the conversion of *hacA^u^* into *hacA^i^,* indicating activation of the UPR under these conditions (Figure 3A). The smaller size of the *hacA^i^* mRNA is consistent with a 5′ mRNA truncation that has been reported following UPR induction in other filamentous fungi [29],[30]. In contrast to wt *A. fumigatus*, the *ΔhacA* mutant was unable to increase the level of three other UPR target genes when treated with DTT, indicating a defect in UPR-regulated gene expression. Complementation of the *ΔhacA* mutant (C') restored UPR signaling to the Δ*hacA* mutant (Figure 3B).

### The UPR Is Required under Conditions of ER Stress

**Figure 4:**
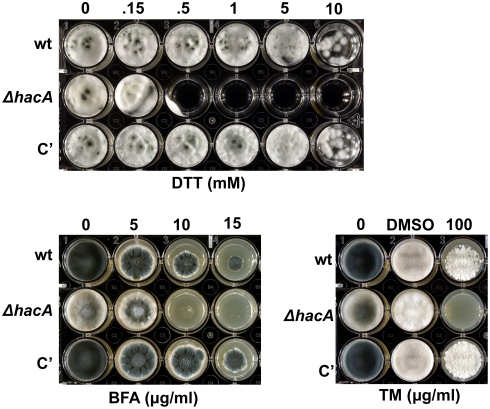
The *ΔhacA* mutant is hypersensitive to ER-stress. ER stress was induced by incubating in the presence of DTT, BFA, or TM. For analysis of DTT sensitivity, conidia from the indicated strains were inoculated into liquid AMM–glucose in a multi-well plate containing the indicated concentrations of DTT and incubated at 37°C for 4 days. For analysis of BFA or TM sensitivity, conidia were inoculated onto solid medium (IMA) in a multi-well plate containing the indicated concentrations of BFA or TM and incubated at 37°C for 2–3 days. DMSO was used as the vehicle control for TM. The experiments were performed three times with similar results.

To determine how loss of *hacA* impacts the growth of *A. fumigatus* under conditions of ER stress, the mutant was incubated in the presence of agents that disrupt ER homeostasis by different mechanisms, including DTT, tunicamycin (TM), and brefeldin A (BFA). DTT unfolds proteins directly by reducing disulfide bonds, TM impairs protein folding by inhibiting N-linked glycosylation, and BFA impairs anterograde protein transport from the ER to the Golgi [26]. The growth of the Δ*hacA* mutant was comparable to wt in the absence of ER stress, although conidiation was decreased on solid medium (Figure 4, columns marked ‘0'). However, the *ΔhacA* mutant was unable to grow in the presence of concentrations of DTT, BFA or TM that could be tolerated by wt *A. fumigatus*, indicating heightened sensitivity to ER stress. The mutant was also hypersensitive to the superoxide-generating agent paraquat (Figure S1), consistent with the adverse effects of oxidative stress on protein folding and ER homeostasis [32].

### The UPR Is Required for Thermotolerance in *A. fumigatus*

**Figure 5:**
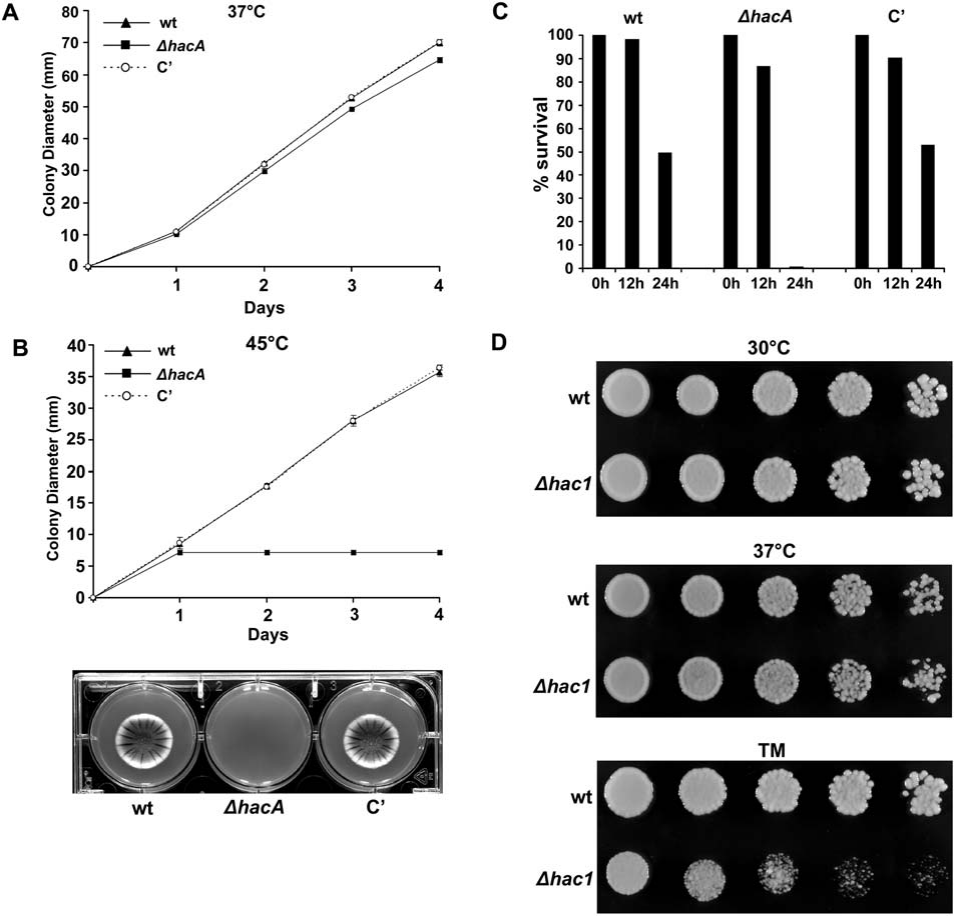
*hacA* is required for growth under thermal stress. Growth of *ΔhacA* at 37°C and 45°C: A 5 µl suspension of conidia from the indicated strains was spotted onto the center of an IMA plate and incubated at 37°C (A) or 45°C (B) for 4 days. The experiment was performed in triplicate, and colony diameter was measured daily. Values represent the mean±SD. Colony morphology after 2 days of growth at 45°C is shown in (B). (C) Reduced survival of Δ*hacA* at 45°C: 200 conidia from the indicated strains were spread in triplicate onto the surface of IMA plates. After incubating at 45°C for 0 h, 12 h, or 24 h, the plates were placed at 37°C and the percentage of surviving CFUs was determined. (D) Temperature sensitivity of *S. cerevisiae Δhac1*: Serial 5-fold dilutions of the wt and the *Δhac1* mutant were spotted onto YPD plates and incubated at 30°C and 37°C for 2 days. Confirmation that the *Δhac1* mutant is hypersensitive to ER stress was shown by inoculating wt and the *Δhac1* mutant onto YPD containing 62.5 ng/ml TM and incubating at 30°C for 2 days. Experiments were performed three times with similar results.

The radial growth rate of the *ΔhacA* mutant was almost indistinguishable from that of wt *A. fumigatus* at 37°C or 42°C (Figure 5A and Figure S2). However, at 45°C the *ΔhacA* mutant failed to grow beyond the site of the initial inoculum (Figure 5B). Microscopic analysis revealed that the *ΔhacA* conidia had germinated at 45°C, but subsequently arrested growth as young hyphae (data not shown). To determine whether this was due to loss of viability, 200 conidia were evenly distributed onto an agar surface. After incubating at 45°C for 0, 12 and 24 h, the plates were shifted to 37°C, and surviving colony forming units (CFUs) were counted. As shown in Figure 5C, approximately 50% of the plated wt and complemented conidia survived 24 h of incubation at 45°C. This was in contrast to the *ΔhacA* mutant, where less than 1% of the plated conidia survived 24 h at 45°C.

*A. fumigatus* is a thermotolerant fungus that normally thrives at temperatures above 50°C [33], with an optimum for growth between 37°C and 42°C, depending on the medium. The *ΔhacA* mutant was unable to grow at a temperature that is only 3°C above the optimum range for this species. This phenotype was unexpected because temperature-induced lethality has not been previously reported in the corresponding *Δhac1* mutant of *S. cerevisiae* [20],[34], suggesting a vulnerability in *A. fumigatus* that may not be present in yeast. *S. cerevisiae* grows optimally between 25°C and 30°C, and 37°C is considered thermal stress for this organism [35]. We found that the growth of a *S. cerevisiae Δhac1* mutant was indistinguishable from that of wt at either 30°C or 37°C, indicating that yeast differ from *A. fumigatus* in their ability to tolerate thermal stress in the absence of proper UPR signaling (Figure 5D).

### The UPR Contributes to Cell Wall Integrity in *A. fumigatus*

**Figure 6:**
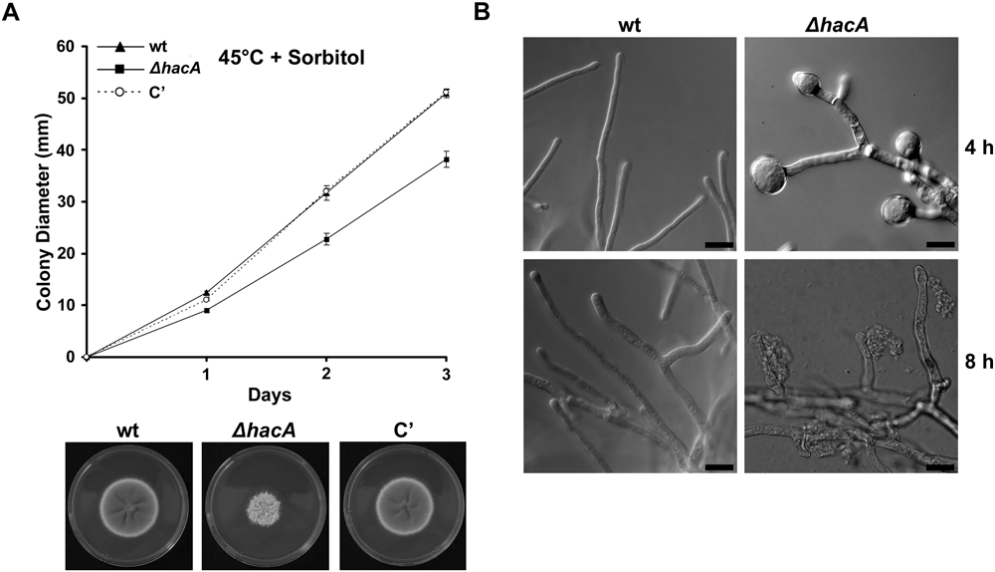
The UPR contributes to cell wall integrity at 45°C. (A) The temperature-sensitivity of *ΔhacA* is remediated by sorbitol: Conidia were inoculated onto the center of an IMA plate containing 1.2 M sorbitol and incubated at 45°C for 3 days. The experiment was performed in triplicate and colony diameter was measured daily. Values represent the mean±SD. Colony morphology after 3 days of incubation at 45°C is shown in the photograph below. (B) Loss of cell wall integrity at 45°C: wild type and *ΔhacA* conidia were inoculated onto coverslips in liquid AMM–glucose and incubated at 37°C for 24 h. The plates were then shifted to 45°C and the hyphae were photographed after 4 and 8 h by DIC microscopy. Experiments in Figure 6 were performed twice with similar results. Scale bar represents 30 µm.

The inability of the *ΔhacA* mutant to grow at 45°C could be reversed by osmotic stabilization of the medium with sorbitol (Figure 6A) or KCl (data not shown), suggesting that the impaired growth of Δ*hacA* at elevated temperature is due, in part, to loss of cell wall integrity. To test this more directly, conidia were inoculated onto cover-slips in liquid medium and germinated overnight at 37°C. After shifting to 45°C, the hyphae were examined microscopically. Within 4 h of incubation at the elevated temperature, the hyphal tips began to swell, and tip lysis became apparent within 8 h (Figure 6B). Occasional areas of cytoplasmic leakage were also observed in subapical hyphae, possibly representing emerging branch points (data not shown). The Δ*hacA* mutant was severely growth impaired following the temperature shift, suggesting an important role for HacA in the maintenance of cell wall integrity at the hyphal tips during thermal stress.

**Figure 7:**
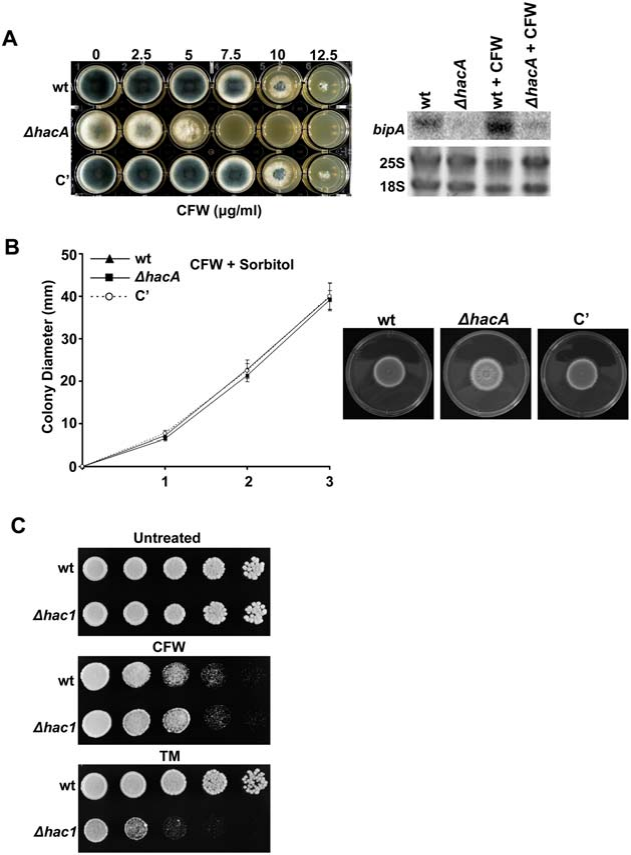
The *ΔhacA* mutant is hypersensitive to cell wall perturbation. (A) CFW sensitivity: conidia were inoculated into a multi-well plate containing IMA supplemented with the indicated concentrations of CFW and incubated for 36 h at 37°C (left panel). Northern blot analysis of *bipA* expression following treatment of overnight cultures with 200 µg/ml of CFW for 1 h is shown in the right panel. (B) CFW sensitivity is remediated by sorbitol: conidia from the indicated strains were spotted in triplicate onto the center of IMA plates containing 10 µg/ml CFW and 1.2 M sorbitol and colony diameter was monitored for 3 d at 37°C. Values represent the mean±SD. (C) *S. cerevisiae Δhac1* is not hypersensitive to CFW. Serial 5-fold dilutions of the wt and the *Δhac1* mutant were spotted onto YPD plates containing 25 µg/ml of CFW and incubated at 30°C for 2 days. Similar results were obtained at concentrations of CFW up to 50 µg/ml (data not shown). A control plate showing the expected hypersensitivity of the *Δhac1* mutant to 62.5 ng/ml TM is shown for comparison. Experiments in Figure 7 were repeated with similar results.

Since thermal stress is likely to have pleiotropic effects on cell physiology, calcofluor white (CFW) was used as a more specific inhibitor of cell wall integrity. CFW is an anionic dye that weakens the wall by binding to nascent chitin chains [36]. Northern blot analysis revealed that treatment with CFW induces the *hacA*-dependent accumulation of *bipA* mRNA (Figure 7A), suggesting that the UPR is part of the normal adaptive response to CFW-induced cell wall stress. The Δ*hacA* mutant was unable to grow in the presence of concentrations of CFW that had minimal effect on the wt or complemented strains, consistent with a protective role for HacA under these conditions (Figure 7A). Normal growth could be restored to the mutant by osmotic stabilization of the medium with sorbitol, supporting the notion that the impaired growth of the mutant in the presence of CFW was a consequence of reduced cell wall integrity (Figure 7B). Microscopic analysis of the Δ*hacA* mutant in the presence of CFW revealed the same apical lysis that was observed under conditions of thermal stress (data not shown), suggesting that the mutant wall is particularly vulnerable to cell wall perturbation at the tips. Similar results were obtained using the related cell wall damaging compound Congo red (Figure S3). However, these findings in *A. fumigatus* were in contrast to *S. cerevisiae*, where the corresponding *Δhac1* mutant showed wt sensitivity to CFW (Figure 7C).

The increased vulnerability of the Δ*hacA* mutant to cell wall stress raises the possibility that HacA contributes to cell wall homeostasis in *A. fumigatus*. To test this, a biochemical analysis of the cell wall was performed. As shown in Table 1, the Δ*hacA* mutant revealed a significant decrease in glucose content in both the alkali insoluble (AI) and alkali soluble (AS) fractions of the cell wall relative to wt, suggesting a defect in both β(1–3) and α(1–3) glucan composition in the mutant cell wall.

**Table 1:** Monosaccharide composition of the alkali-insoluble and alkali-soluble fractions of the cell wall from the wt and Δ*hacA* strains

	Alkali-insoluble (AI)		Alkali-Soluble (AS)	
	wt	Δ*hacA*	wt	Δ*hacA*
Mannose	6.4±0.2	4.5±1.7	1.0±0.2	1.0±0.5
Glucose *	47.0±3.0	33.0±3.5	66.0±3.0	52.0±4.0
Galactose	6.6±1.0	6.5±0.5	1.7±0.7	4.5±0.7
N-acetylglucosamine	24.0±2.0	21.0±1.8	-	-
N-acetylgalactosamine	1.0±1.0	3.8±1.3	1.2±0.9	2.5±1.0

Results expressed as percent – average of four replicates±standard deviation.

*: Statistically significant (*P*<0.001).

The ratio of AI/AS for wt and Δ*hacA* was not significantly different (1.6±0.3 and 1.3±0.2 for wt and Δ*hacA*, respectively).

### Loss of UPR Function Enhances Susceptibility to Antifungal Drugs

**Figure 8:**
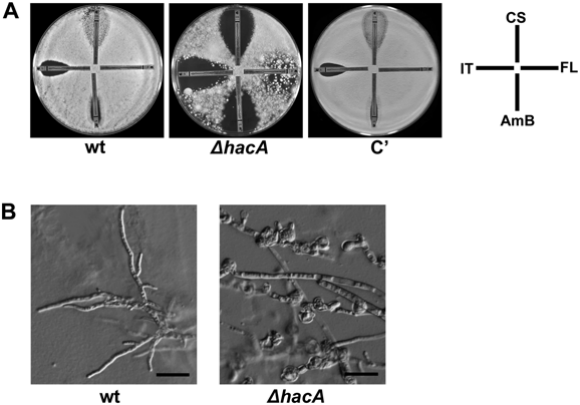
The *ΔhacA* mutant is hypersensitive to antifungal drugs. (A) Antifungal susceptibility using Etest: conidia were spread evenly onto a 150 mm plate of RPMI agar. Four Etest strips, each impregnated with a concentration gradient of caspofungin (CS), fluconazole (FL), amphotericin B (AmB), and itraconazole (IT) were applied to the surface, with the highest concentration oriented at the plate edge. (B) Microscopic analysis of caspofungin-treated hyphae. Equal numbers of conidia from the indicated strains were spread onto the surface of an IMA plate and allowed to germinate at 30°C for 24 h. A caspofungin Etest strip was then applied to each plate and incubated overnight at 37°C. The morphology of the hyphae surrounding the highest concentration on the strip was observed by DIC microscopy. Scale bar represents 50 µm.

All major classes of antifungal drugs that are currently in use against *A. fumigatus* attack the integrity of the membrane or cell wall. Fungi respond to these agents by upregulating cell wall and membrane repair systems [37],[38],[39], which may increase stress on the secretory system. To determine how loss of UPR function would affect growth in the presence of antifungal stress, susceptibility to amphotericin B, caspofungin, itraconazole and fluconazole was compared using the Etest method. Conidia were spread onto the surface of an agar plate, and 4 Etest strips were placed on top, each impregnated with a concentration gradient of a different antifungal drug, and incubated at 37°C for 48 h. The *ΔhacA* mutant had larger areas of growth inhibition surrounding each strip, indicating heightened susceptibility to each of these drugs and a decrease in the minimal inhibitory concentration (Figure 8A). The incomplete clearing around the caspofungin strip on plates inoculated with the wt or complemented strains is consistent with the known fungistatic activity of this class of drug for *A. fumigatus* [40]. It is therefore striking that a complete zone of clearing was evident around the caspofungin strip on the plate inoculated with the *ΔhacA* mutant. Agar plugs taken from the zone of growth inhibition surrounding the caspofungin strip on wt-inoculated plates were able to grow when transferred to medium lacking any drug. However, no viable organism could be recovered from agar plugs taken from the cleared zone surrounding the caspofungin strip on *ΔhacA*-inoculated plates, indicating that caspofungin becomes fungicidal in the absence of UPR function. Microscopic analysis of caspofungin-treated hyphae revealed normal morphology in the wt, but abnormal swelling and lysis in the Δ*hacA* mutant (Figure 8B). These defects were localized to hyphal tips and branch points, similar to what was observed under conditions of thermal stress and CFW treatment. This experiment was performed on RPMI agar in accordance with the manufacturer's specifications, but comparable results were also obtained using IMA as the medium (Figure S4). Remarkably, the corresponding *Δhac1* mutant in *S. cerevisiae* did not show increased sensitivity to either caspofungin, ketoconazole, amphotericin B or fluconazole (Figure S5).

### The UPR Supports Protease Secretion and Growth on Complex Substrates

**Figure 9:**
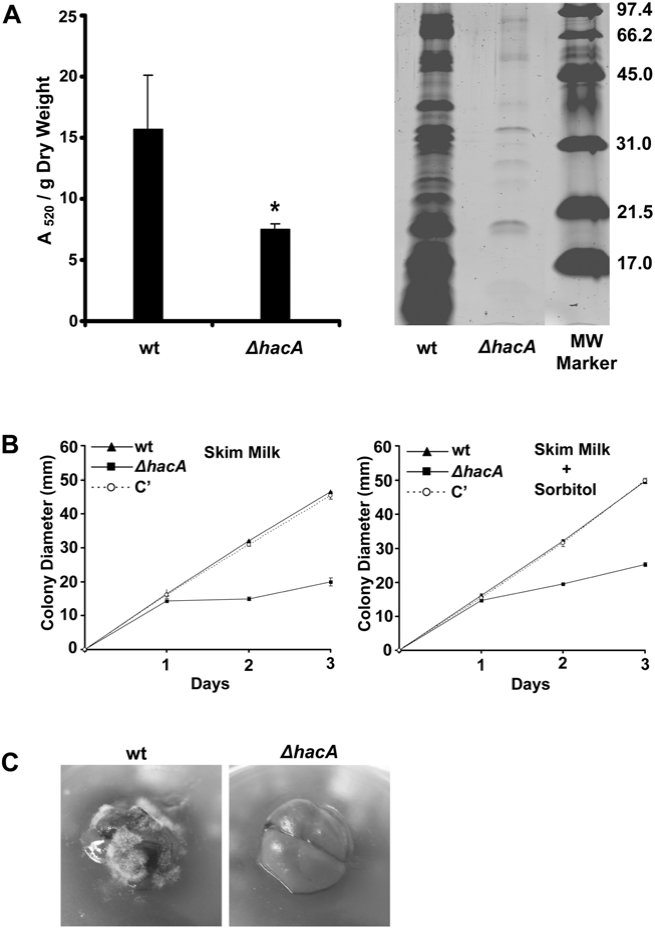
The UPR supports protease secretion and growth on complex substrates. (A) Azocoll hydrolysis by *A. fumigatus* proteases: Equal numbers of conidia were inoculated in liquid AMM-FBS, and incubated at 37°C/150 rpm for 72 h. Culture supernatants were incubated in the presence of Azocoll for 3 h and the absorbance of the medium at 520 nm was determined. The experiment was performed in triplicate and values represent the mean A_520_/g dry weight±SD. Significance was assessed using a two-tailed t-test, and the asterisk indicates that the Azocoll hydrolysis is significantly less in the *ΔhacA* mutant than in the wt (p<0.05). 1D gel analysis of wt and Δ*hacA* secreted proteins: Equal numbers of wt and Δ*hacA* conidia were inoculated into liquid AMM and incubated for 3 days at 37°C without shaking. The hyphal mat was removed, and the supernatant was concentrated as described in Materials and Methods. A 5 µl aliquot of each sample was then fractionated by 1D SDS-PAGE and proteins were stained by SYPRO Ruby dye. The protein marker is shown in kD. (B) Growth on skim milk: Conidia were spotted onto skim milk agarose plates in the presence or absence of 1.2 M sorbitol and colony diameter was monitored daily at 37°C. The experiment was performed twice, with similar results. (C) Growth on mouse lung tissue: A freshly isolated piece of mouse lung tissue was inoculated with wt or *ΔhacA* conidia and incubated at 37°C for 24 h. Experiments in Figure 7 were repeated with similar results.

The ability of *A. fumigatus* to colonize the host begins with the germination of conidia in the lung followed by invasion of exploring hyphae into the surrounding tissue. The organism must acquire nutrients from host tissues at all steps of the infection, which requires continual secretion of a multitude of degradative enzymes. Since ER stress occurs when protein secretion is upregulated [41], we hypothesized that loss of UPR signaling would impair the secretory capacity of *A. fumigatus*. To test this prediction, secreted proteolytic activity was quantified with the Azocoll assay, using conditions previously described for *A. fumigatus* [10]. Azocoll is an insoluble collagen linked to an azo dye, and its hydrolysis releases soluble colored peptides that can be quantified colorimetrically [42]. As shown in Figure 9A, culture supernatants derived from the *ΔhacA* mutant were significantly less efficient at digesting Azocoll than wt cultures, indicating that protease secretion is abnormal in the mutant. This decrease in proteolytic activity was consistent with an overall reduction in secreted protein levels in the Δ*hacA* mutant, as revealed by SDS-PAGE analysis of culture supernatants (Figure 9A).

The reduced secretory capacity of the *ΔhacA* mutant predicts that this strain would have difficulty assimilating nutrients from a complex substrate. On IMA medium, the growth of the *ΔhacA* mutant was normal (Figure 5A). However, this rich medium contains a substantial amount of reduced carbon and nitrogen in the form of tryptone (pancreatic digest of casein), peptone (enzymatic digest of proteins), yeast extract, dextrose and starch. When challenged to use a more complex substrate such as skim milk, the *ΔhacA* mutant grew slower than the wt and complemented strains (Figure 9B). Osmotic stabilization with sorbitol was unable to rescue this phenotype, but the addition of a reduced nitrogen/carbon source completely restored growth to wt levels (Figure 9B, and data not shown). These findings argue that the impaired growth of *ΔhacA* on skim milk agar is due to inefficient nutrient acquisition rather than an indirect effect on cell wall stress. Similar observations were made when mouse lung tissue was used as a substrate. In contrast to the wt-inoculated lung tissue, which supported fungal growth within 24 h, the *ΔhacA-*inoculated lung showed no signs of fungal growth (Figure 9C). Collectively, these findings suggest that the UPR promotes the growth of *A. fumigatus* on complex polymeric material by facilitating the production of secreted hydrolases that are necessary to breakdown the substrate into usable nutrients.

### The UPR Promotes Virulence of *A. fumigatus*

**Figure 10:**
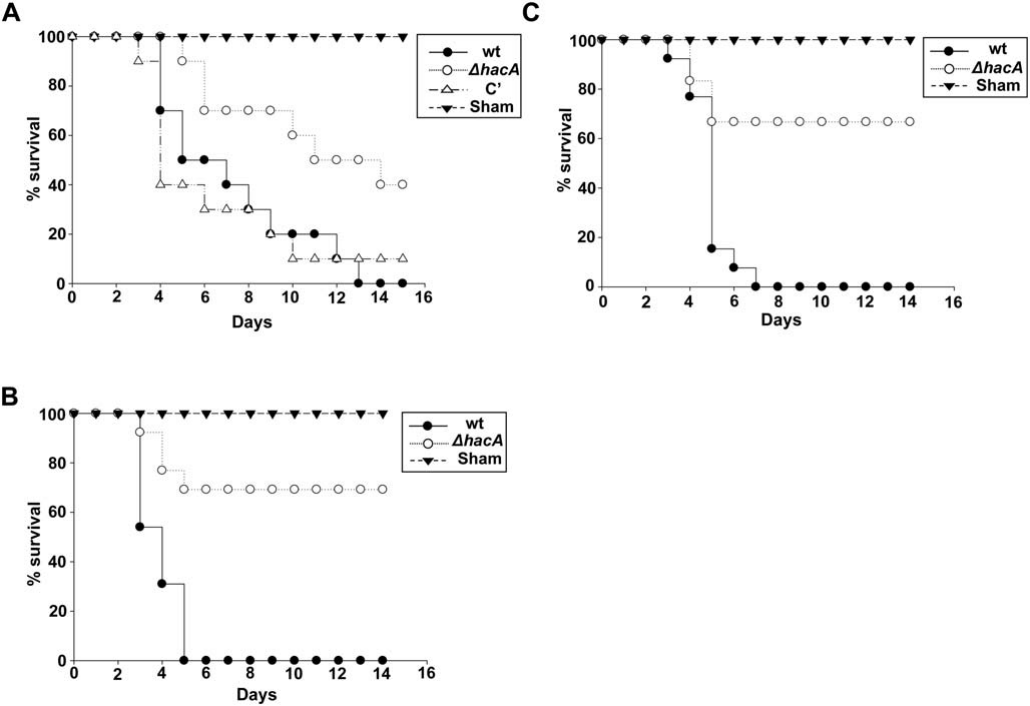
The *ΔhacA* mutant has attenuated virulence in three models of invasive aspergillosis. (A) Triamcinolone immunosuppression model: CF-1 outbred mice were immunosuppressed with triamcinolone acetonide and infected intranasally with conidia from the indicated strains. Mortality was monitored for 15 days. (B) Cortisone acetate immunosuppression model: C57BL/6 mice were immunosuppressed with cortisone acetate and infected intratracheally with conidia from the wt or the *ΔhacA* mutant. Mortality was monitored for 14 days. (C) Neutropenia model: C57BL/6 mice were immunosuppressed using a combination of the antibody RB6-8C5 and cyclophosphamide. The mice were infected intratracheally with the wt or the *ΔhacA* mutant, and mortality was monitored for 14 days.

The ability to detect *A. fumigatus* proteases *in vivo* [43] implies that active secretion occurs in the host environment, suggesting that the UPR may contribute to virulence. To test this, we compared the virulence of the *ΔhacA* mutant to that of wt *A. fumigatus*. As shown in Figure 10A, the *ΔhacA* mutant was hypovirulent in an outbred mouse model of invasive aspergillosis that uses a single dose of triamcinolone acetonide (TA) to induce a period of transient immunosuppression. An increasing body of evidence suggests that the outcome of virulence testing in experimental models of aspergillosis is influenced by host strain and the type of immunosuppression [44],[45],[46]. Thus, virulence was also compared in two additional models that use inbred mice: a neutropenic model and a cortisone acetate model. The *ΔhacA* mutant had attenuated virulence in all three model systems, demonstrating that the UPR is an important stress signaling pathway in the host environment (Figure 10A, 10B, and 10C).

## Discussion

All eukaryotes with an elevated capacity for protein production depend on the UPR to maintain ER homeostasis. ER stress is encountered under many adverse environmental conditions that cause protein unfolding, including high temperature [47],[48], oxidative stress [49],[50], hypoxia [51],[52] or nutrient limitation [53]. However, it may also occur under normal physiological conditions in response to a change in the demand for secretion. For example, the UPR is induced in B cells when they are stimulated to secrete antibody [54]. Similarly, UPR-deficient mice fail to differentiate hepatocytes, pancreatic β cells or plasma cells, because the UPR protects against the ER stress that is generated when the intense secretory activity of these cells is activated [55],[56],[57]. *A. fumigatus,* like many other filamentous fungi, is well equipped for protein secretion [5],[9],[58], with over 1% of its genome dedicated to secreted proteases alone [59],[60]. This robust secretory arsenal makes filamentous fungi excellent production hosts for proteins of biotechnology interest [11],[12],[13],[61], and the ability of enforced HacA^i^ overexpression to further enhance protein secretion [62] illustrates the importance of the UPR to the maintenance of ER homeostasis under a high secretory load.

In this study, a UPR-deficient mutant of *A. fumigatus* was constructed in order to determine how the UPR impacts growth, secretion and virulence in *A. fumigatus*. The *ΔhacA* mutant was hypersensitive to agents that perturb ER homeostasis and was unable to increase the expression of four known UPR target genes in response to ER stress. Although a *hacA*-independent mechanism of *bipA* induction has been recently reported in *A. niger* strains that overproduce membrane proteins [63], the absence of *bipA* induction in the *ΔhacA* mutant treated with DTT indicates that *bipA* induction is *hacA*-dependent when DTT is used to induce ER stress.

In *S. cerevisiae*, *Δhac1* mutants are inositol auxotrophs, a phenotype that is associated with defects in expression of *INO1* [34]. However, inositol was dispensable for the growth of the *A. fumigatus ΔhacA* mutant (data not shown), indicating that *hacA* is not required for this pathway in *A. fumigatus*. On rich medium, the growth of the *ΔhacA* mutant was comparable to that of wt. However, the *ΔhacA* mutant became growth impaired when it was forced to obtain nutrients from complex substrates such as skim milk or mouse lung tissue. These findings argue that a rapid growth rate *per se* does not constitute sufficient ER stress to require extensive support from the UPR, as long as an adequate supply of reduced nitrogen/carbon is present. By contrast, growth on more polymeric material would require an increase in secretory activity to breakdown the substrate, resulting in UPR activation. Communication from other stress response pathways may also influence the magnitude of this response, such as the ability of Gcn4p to control both amino acid starvation responses and UPR target gene expression [64]. Failure to trigger the UPR under situations that demand increased secretory capacity would be expected to impair secretion, thereby limiting nutrient availability and reducing growth. Furthermore, the unresolved ER stress caused by loss of UPR function may trigger a second feedback mechanism that is activated in response to impaired secretory protein folding or transport called repression under secretion stress (RESS) [65]. RESS involves the selective transcriptional downregulation of genes encoding certain secreted proteins, and current evidence suggests that it is controlled differently from the UPR [66]. Our finding of reduced secreted collagenolytic activity and an alteration in the overall secretory profile of the *ΔhacA* mutant is consistent with this model (Figure 9A).

In nature, *A. fumigatus* has evolved to thrive in compost, an environmental niche that generates heat from microbial activity. *A. fumigatus* has acquired unique mechanisms of thermotolerance to support its growth up to 60°C [33]. This study demonstrates that the UPR plays an essential role in thermotolerance. The *ΔhacA* mutant grew normally at 37°C but was unable to maintain cell wall integrity at 45°C. Cytoplasmic leakage was observed at hyphal tips and at various points along the hypha, possibly representing areas of weakness caused by dynamic remodeling of the cell wall at these sites. Reduced thermotolerance has also been reported in other cell wall mutants of filamentous fungi, suggesting that thermal stress has adverse effects on the cell wall [67],[68]. Prolonged incubation of the Δ*hacA* mutant at 45°C was incompatible with viability. This finding is particularly notable in view of the extraordinary thermotolerance of *A. fumigatus*, but is also remarkable because the corresponding mutation in *S. cerevisiae* does not exhibit the same temperature-sensitive phenotype (Figure 5). This difference may reflect the need for more surface export functions in A. fumigatus to maintain the integrity of the apical cell wall during polarized growth, particularly at elevated temperature.

Direct perturbation of the cell wall by treatment with CFW increased apical lysis and reduced hyphal growth in the Δ*hacA* mutant. Sorbitol rescued this CFW sensitivity, suggesting that the phenotype is primarily a consequence of reduced cell wall integrity (Figure 7B). By contrast, neither the temperature sensitivity nor the reduced conidiation of the *ΔhacA* mutant could be fully rescued by sorbitol (Figure 6A), suggesting that the UPR has additional homeostatic functions under conditions of thermal stress that do not involve the cell wall. The components of the *A. fumigatus* cell wall can be divided into two main groups based on their alkali solubility [69]. The AI fraction is thought to provide the main structural rigidity of the wall and is composed of β(1–3) glucan, chitin and galactomannan. By contrast, the AS fraction contains predominantly α(1–3) glucan and galactomannan. The heightened sensitivity of the Δ*hacA* mutant to multiple types of cell wall stress, combined with the decreased glucose content in its cell wall, is consistent with a defect that reduces the overall glucan composition of the cell wall. The *A. fumigatus* wall is a highly dynamic structure, particularly at hyphal tips and branch points where the structural needs of the hypha must be balanced by the demand for new apical growth [69]. Since β(1,3) glucan synthase is transported to the growing tips as an inactive complex through the secretory pathway [70], it is intriguing to speculate that the predisposition of the Δ*hacA* mutant to apical lysis is due to inefficient delivery of the glucan synthase complex to the growing tips. The Δ*hacA* mutant had normal chitin levels however, suggesting that HacA is less important for chitin synthase activity. This may reflect the ability of multiple chitin synthases [71] to compensate for any reduction in chitin synthase delivery caused by loss of HacA. Interestingly, the *S. cerevisiae Δhac1* mutant had wt sensitivity to CFW, suggesting a fundamental difference between these two species in terms of their reliance on the HacA-dependent UPR for cell wall homeostasis.

Analysis of the antifungal susceptibility profile of the *ΔhacA* mutant revealed two important findings. First, the *ΔhacA* mutant showed a dramatic increase in susceptibility to antifungal drugs that are in use for the treatment of invasive aspergillosis. This suggests that targeting the UPR with novel therapy could act synergistically with currently approved antifungal drugs, as well as potentially increasing the susceptibility profile of other fungal pathogens that are intrinsically resistant to some antifungals. For example, *A. terreus* and most *Fusarium* and *Scedosporium* isolates are only moderately susceptible or resistant to amphotericin B [72],[73],[74],[75]. Similarly, itraconazole has limited activity against *Fusarium* and *Scedosporium* species, and voriconazole and echinocandins are largely ineffective against zygomycetes [72],[76]. Thus, targeting the UPR has the possibility of expanding the number of therapeutic options for these emerging fungal pathogens. The second important observation is that the well known fungistatic effects of the β(1–3) glucan synthase inhibitor caspofungin became fungicidal to *A. fumigatus* in the absence of *hacA* function. This synergistic activity is likely to reflect the lethal effects of glucan synthase inhibition in a strain that is already deficient in glucan production. These observations also suggest that the UPR is an essential component of the adaptive response to antifungal stress, an idea that is supported by the upregulation of genes involved in ER and secretion functions, including *Hac1*, during caspofungin treatment of the dimporphic yeast *Candida albicans* [77]. This class of genes was not induced by caspofungin treatment of *S. cerevisiae*, suggesting an important difference between these yeasts [39],[78]. In addition, loss of UPR function increases sensitivity to cell wall damage in *A. fumigatus* (Figure 5) and in *C. albicans* [79], but not in *S. cerevisiae* (Figure 5). One possible explanation for these differences is that the ability to form true hyphae in *A. fumigatus* and *C. albicans* increases the demand on the secretory system for cell wall repair, making these fungi more vulnerable to loss of UPR function.

Since recently published data have shown that the virulence of *A. fumigatus* is influenced by host strain and the type of immunosuppression, three distinct mouse models of invasive aspergillosis were used to assess virulence. The *ΔhacA* mutant was hypovirulent in all three models, emphasizing the importance of UPR signaling to the ability of the fungus to grow in the host environment. Metabolic evidence has suggested that *A. fumigatus* relies heavily on protein degradation as a major source of nutrients *in vivo* [80], which is consistent with the detection of secreted *A. fumigatus* proteases *in vivo* [43]. Although protease secretion has long been considered a virulence-related factor for *A. fumigatus*, single-gene disruptions have yet to demonstrate this because of the abundant secreted proteases encoded by the genome. Here, we provide the first genetic evidence to suggest that secretory activity is important to the virulence of this organism. The results are consistent with a model in which the UPR contributes to virulence by supporting the secretory activity that is necessary to degrade host tissues. In the absence of a functional UPR, this secretory capacity is impaired, which may lessen the ability of the organism to damage tissues and efficiently extract the nutrients required for growth. Failure to resolve ER stress could also contribute to the reduced virulence of the Δ*hacA* mutant if unfolded proteins accumulate to toxic levels.

Taken together, the data from this study suggest that the high secretory capacity of *A. fumigatus* places it at considerable risk for ER stress and thus represents a vulnerability that could be exploited for therapeutic gain by disrupting the pathways that maintain ER homeostasis. Moreover, since secretory processes have prominent roles in the virulence of parasitic protozoa [81],[82], the findings from this study may have relevance to other pathogenic eukaryotes.

## Materials and Methods

### Strains and Culture Conditions

The *A. fumigatus* and *S. cerevisiae* strains used in the study are listed in Table 2. Conidia were harvested from *Aspergillus* minimal medium (AMM) [83] containing 10 mM ammonium tartrate and osmotically stabilized with 1.2 M sorbitol. Unless otherwise specified, experiments involving the *ΔhacA* mutant were performed on inhibitory mold agar (IMA, Fisher Scientific Cat. # 14-910-95) since the growth rate of the *ΔhacA* mutant approximated that of wt on this medium. Radial growth rate was determined by spotting 5,000 conidia onto the center of a plate and monitoring colony diameter daily. For analysis of survival under thermal stress, 200 conidia were evenly spread onto the surface of an IMA plate. After incubating at 45°C for 0 h, 12 h, or 24 h, the plates were placed at 37°C and surviving colony forming units (CFUs) were counted after 24 h of growth. To demonstrate cytoplasmic leakage at 45°C, conidia were inoculated onto a glass coverslip submersed in AMM and incubated at 37°C for 24 h. After shifting to 45°C for 4 h and 8 h, the coverslip was inverted onto a glass slide and the hyphae were photographed by differential interference contrast (DIC) microscopy.

**Table 2:** Strains used in this study

Strain	Species	Genotype	Source
WT (*ΔakuA)*	*A. f.*	*akuA*::*ptrA*	S. Krappmann
*ΔhacA*	*A. f.*	*akuA*::*ptrA, hacA*::*hph*	This study
C'	*A. f.*	*ΔhacA (hacA/ble)*	This study
BY4741	*S. c.*	MAT**a**; *his3Δ1*; *leu2Δ0*; *met15Δ0*; *ura3Δ0*	Invitrogen
BY4741 (*Δhac1*)	*S. c.*	MAT**a**; *his3Δ1*; *leu2Δ0*; *met15Δ0*; *ura3Δ1*, Δ*hac1::kan*	Invitrogen

To monitor growth under ER or cell wall stress, 2,000 conidia were inoculated onto the center of a plate containing IMA supplemented with concentrations of BFA, TM, CFW, or Congo red specified in the Results section. The plates were incubated for 2–3 days at 37°C, and the extent of growth was used as a relative indicator of sensitivity. Since it is recommended that DTT-induced ER stress be performed in liquid rather than solid medium [26], analysis of DTT sensitivity was performed by inoculating 10,000 conidia in liquid AMM containing the indicated concentrations of DTT and incubating at 37°C for 4 days. Utilization of skim milk was determined by inoculating 5,000 conidia onto skim milk agarose plates (0.5% skim milk, 0.8% agarose), or skim milk agarose supplemented with 1.2 M sorbitol. The plates were incubated at 37°C and radial growth was monitored daily for 3 days.

For experiments involving *S. cerevisiae*, overnight cultures of the wt and the *Δhac1* mutant (Invitrogen) were diluted to an OD_600_ of 0.1 and cultured at 30°C and 250 rpm until the OD_600_ reached 0.5. Serial 5-fold dilutions were then spotted onto YPD (1% yeast extract, 2% peptone, 2% glucose) plates containing TM (62.5 ng/ml) or CFW (25–50 µg/ml), and the plates were incubated at 30°C or at 37°C for 2 days.

### Antifungal Susceptibility

Antifungal susceptibility of *A. fumigatus* strains was determined using the Etest diffusion assay (AB BIODISK) according to the manufacturer's instructions. Briefly, conidial suspensions were prepared in sterile distilled water and adjusted to 1×10^6^ conidia/ml. One ml of the conidial suspension was then spread evenly onto the surface of a 150 mm plate of RPMI agar buffered with MOPS (Remel, Lenexa, Kansas), using a glass spreader. The inoculated agar surface was allowed to dry for 20 min before Etest strips containing amphotericin B, caspofungin, itraconazole, fluconazole or ketoconazole were applied. The plates were incubated at 37°C for 48 h before being photographed. The MICs were read as the lowest drug concentrations at which the border of the elliptical inhibition zone intercepted the scale on the antifungal strip. For Etest experiments involving *S. cerevisiae* strains, overnight cultures in YPD were diluted to an OD_600_ of 0.1 and cultured at 30°C /250 rpm until the OD_600_ reached 0.5 . The cultures were diluted to an OD_600_ of 0.257 and the yeast were spread evenly onto the surface of duplicate YPD plates using a Q-tip. Etest strips were applied after allowing the plates to dry for 20 min, and the plates were incubated for 48 h at 30°C.

### Deletion and Reconstitution of the *A. fumigatus hacA* Gene

All PCR primers used in the study are listed in Table 3. The *A. fumigatus hacA* gene (Genbank accession XM_743634) was disrupted using the split-marker approach [84]. The left arm of *hacA* was PCR amplified from genomic DNA using PFU turbo polymerase (Stratagene) with primers 522 and 523 creating PCR product #1. The first two thirds of the hygromycin resistance cassette was amplified from plasmid pAN7-1 using primers 395 and 398 to create PCR product #3. PCR products #1 and #3 were then combined in an overlap PCR reaction with primers 395 and 522 to generate PCR product #5. PCR product #5 was then cloned into pCR-Blunt II- TOPO (Invitrogen) to create p527. The right arm of the *hacA* gene was then PCR amplified from genomic DNA using primers 524 and 525 to generate PCR product #2. The second two-thirds of the hygromycin resistance cassette was amplified from pAN7-1 with primers 396 and 399 to make PCR product #4, and PCR products #2 and #4 were combined in an overlap PCR reaction with primers 396 and 525 to generate PCR product #6. PCR product #6 was then cloned into pCR-Blunt II- TOPO to create p528. The inserts from p527 and p528 were gel-purified following digestion with *BstX1* and *Smal*, and 10 µg of each was used to transform wt-Δ*akuA* protoplasts as previously described [85]. Inositol was included into the selection plates since *S. cerevisiae* UPR mutants are inositol auxotrophs [34]. Hygromycin-resistant colonies were screened by PCR, and loss of the *hacA* gene was confirmed on monoconidial isolates by genomic Southern blot analysis as described in the results section. Probe A was PCR-amplified from wt genomic DNA using primers using primers 492 and 493 while probe B was amplified with primers 522 and 523. Genomic Southern blot genotyping confirmed single-copy deletion of the *hacA* gene in 2 transformants, and these clones were used for phenotypic analysis.

**Table 3:** PCR primers used in this study. M13-derived sequences used for overlap PCR are underlined

Primer	Gene	Sequence (5′-3′)
395	*hph*	CTCCATACAAGCCAACCACGG
396	*hph*	CGTTGCAAGACCTGCCTGAA
398	*hph*	CGCCAGGGTTTTCCCAGTCACGACAA GTGGAAAGGCTGGTGTGC
399	*hph*	AGCGGATAACAATTTCACACAGGA TCGCGTGGAGCCAAGAGCGG
492	*hacA*	TGCGATAGACGCTGGAGAAG
493	*hacA*	CATCACGCCTACGAAATGGA
494	*bipA*	GTCTGATTGGACGCAAGTTC
495	*bipA*	ATCTGGGAAGACAGAGTACG
522	*hacA*	CCTTCGCTACAGACACATGG
523	*hacA*	GTCGTGACTGGGAAAACCCTGGCG CCGGTAGACAAGATCACAGG
524	*hacA*	TCCTGTGTGAAATTGTTATCCGCT ATTGCAGCTGGCTGTTAGTG
525	*hacA*	CCTCTATCGCACTACTAGCG
572	*hacA*	TCGCTCGAATTTCGCGAAGA
602	*pdiA*	ATGCGGTCTTTTGCTCCCTTG
603	*pdiA*	TACCGGCCGTAATATAGCTAG
628	*tigA*	ATGGCTCGGTTGAGCTTCCTG
629	*tigA*	TTATAGCTCGTCCTTGGCATT

To construct the complementation plasmid, a phleomycin resistance cassette was excised from plasmid pBCphleo as a *Sal*I/*Hind*III fragment and ligated into the *Xho*I/*Hind*III sites of plasmid pSL1180. The *trpC* terminator was PCR-amplified from plasmid pAN7-1 and cloned into the vector as a *Sac*II/*Sac*I fragment to create plasmid pTPP. The *hacA* gene containing 1128 bp upstream of the predicted translational start site was PCR-amplified from wt genomic DNA using primers 522 and 527 and cloned into pCR-Blunt II-TOPO. The *hacA* gene fragment was excised from pCR-Blunt II- TOPO with a *BamHI* and *NotI* restriction digest and inserted into the *Bgl*II/*Not*I sites of pTPP. Ten µg of the plasmid was linearized with *ApaI* and transformed into *ΔhacA* mutant protoplasts as previously described [85]. Southern blot analysis of phleomycin-resistant colonies revealed that the complementation plasmid integrated homologously at the *hacA* locus,and at least one ectopic site (data not shown).

### Northern Blot Analysis and cDNA Cloning

Total RNA was extracted from overnight cultures by crushing the mycelium in liquid nitrogen and resuspending in TRI reagent LS (Molecular Research Center, Cincinnati, OH). The RNA was fractionated by formaldehyde gel electrophoresis and ribosomal RNA (rRNA) loading was visualized by SYBR-Green II staining and quantified using a STORM phosphorimager (Molecular Dynamics). The RNA was transferred to BioBond nylon membranes (Sigma), and hybridized to a ^32^P-labeled DNA probe for *A. fumigatus bipA, pdiA, tigA or hacA*. The *bipA* fragment was PCR-amplified from wt *A. fumigatus* genomic DNA using primers 494 and 495, the *pdiA* fragment was amplified using primers 602 and 603, the *tigA* fragment was amplified using primers 628 and 629, and the *hacA* fragment was amplified with primers 492 and 493. Hybridization intensities were quantified by Phosphorimager analysis and normalized against SYBR®-Green II-stained rRNA intensity.

The uninduced form of the *hacA* mRNA (*hacA^u^*) was obtained by extracting RNA from overnight cultures of *A. fumigatus*, and the induced form of the *hacA* mRNA (*hacA^i^*) was obtained by extracting RNA from overnight cultures that were treated for 1 h with 1 mM dithiothreitol (DTT). Confirmation that these conditions differentially modulated the conversion of *hacA^u^* to *hacA^i^* was obtained by Northern blot analysis prior to reverse transcription (Figure 3A). The RNA was then reverse-transcribed using the Superscript III reverse transcriptase first-strand synthesis system (Invitrogen) and primers 493 and 572. The resulting cDNAs were cloned into pCR-Blunt II-TOPO® and sequenced.

### Analysis of Protein Secretion

Protease secretion in *A. fumigatus* was quantified using Azocoll (Calbiochem) hydrolysis as previously described [10]. Azocoll is an insoluble collagen linked to a red azo dye, and the release of the dye is indicative of collagen hydrolysis. Conidia were inoculated at a concentration of 1×10^5^/ml in 50 ml of AMM-FBS (*Aspergillus* minimal medium containing 10% heat-inactivated fetal bovine serum (FBS) as the nitrogen and carbon source) [10]. The cultures were incubated at 37°C with gentle shaking at 150 rpm. After 72 h, a 1ml aliquot of the culture was microfuged at 15,000g for 5 min, and a 15 µl aliquot of the supernatant was added to 2.4 ml of a 5 mg/ml suspension of pre-washed Azocoll (prepared by washing and resuspending the collagen particles in buffer containing 50 mM Tris (pH 7.5), 1mM CaCl_2_, and 0.01% sodium azide). The collagen/supernatant mixture was incubated at 37°C for 3 h, with constant shaking at 350 rpm. The Azocoll/supernatant mixture was centrifuged at 13,000 g for 5 min and the release of the azo dye was determined by measuring the absorbance at 520 nm. Values were normalized to the lyophilized weight of the 72 h biomass.

For analysis of total protein secretion, 2.5×10^7^ conidia were inoculated into a 500 ml flask containing 100 ml of AMM and incubated for 3 d at 37°C without shaking. Under these conditions, the wt and Δ*hacA* strains generated a similar amount of dried biomass. The supernatant was removed from both cultures and concentrated to approximately 500 µl using the Amicon 8050 ultrafiltration system with a membrane cut-off of 10 kD. An equal volume of water was added to each sample and further concentrated to 100 µl using an Amicon Ultra Centrifugal Filter Device. Protein concentrations were determined using the Bradford assay and samples were stored at −80°C prior to analysis. For 1D gel analysis, each sample was mixed with sample buffer (200 mM Tris-HCl pH 6.8, 50% glycerol, 5% sodium docecyl sulfate (SDS), 0.5% bromophenol blue and 5% (v/v) β-mercaptoethanol), heated to 95°C and 5 µl of each sample was loaded onto a 12% SDS PAGE gel. Gels were run at 150 V for ∼2 h. After fixing for 30 min in 10% methanol and 7% acetic acid, gels were stained overnight with SYPRO Ruby fluorescent dye, destained for 30 min in fixing solution, then washed with water for 5–10 min prior to imaging with a GE Healcare Typhoon scanner.

### Cell Wall Analysis

Mycelial cell wall fractionation was performed according to the method described by Fontaine *et al.* [86] with slight modification. Briefly, wt and Δ*hac1* strains were grown in a 1.2-liter fermenter in liquid Sabouraud medium. After 24 h of cultivation (linear growth phase), the mycelia were collected by filtration, washed extensively with water and disrupted in a Dyno-mill (W. A. Bachofen AG, Basel, Switzerland) cell homogenizer using 0.5-mm glass beads at 4°C. The disrupted mycelial suspension was centrifuged (3,000×*g* for 10 min), and the cell wall fraction (pellet) obtained was washed three times with water, subsequently boiled in 50 mM Tris-HCl buffer (pH 7.5) containing 50 mM EDTA, 2% SDS and 40 mM β-mercaptoethanol (β-ME) for 15 min, twice. The sediment obtained after centrifugation (3,000×g, 10 min) was washed five times with water and then incubated in 1 M NaOH containing 0.5 M NaBH_4_ at 65°C for 1 h, twice. The insoluble pellet obtained upon centrifugation of this alkali treated sample (3,000×g, 10 min, AI-fraction) was washed with water to neutrality, while the supernatant (AS-fraction) was neutralized and dialyzed against water. Both fractions were freeze-dried and stored at −20°C until further use. Hexose composition in the samples were estimated by gas-liquid chromatography using a Perichrom PR2100 Instrument (Perichrom, Saulx-les-Chartreux, France) equipped with flame ionization detector (FID) and fused silica capillary column (30 m×0.32 mm id) filled with BP1, using meso-inositol as the internal standard. Derivatized hexoses (alditol acetates) were obtained after hydrolysis (4N trifluoroacetic acid/8N hydrochloric acid, 100°C, 4 h), reduction and peracetylation. Monosaccharide composition (percent) was calculated from the peak areas with respect to that of the internal standard.

### Mouse Models of Invasive Aspergillosis

For the cortisone acetate (CA) and cyclophosphamide (CPS) immunosuppression models, cultures were grown for 14 d on IMA agar (Difco) at 25°C. Conidia were collected by washing the agar surface with phosphate buffered saline (PBS) containing 0.05% Tween 20. The conidial suspension was filtered first through sterile gauze and then through a 12 µm filter (Millipore) before washing twice with PBS/Tween. Female 4–6 week old C57BL/6 mice were used in all experiments, with the exception of the triamcinolone model. Inoculum sizes were selected on the basis of pilot experiments with the different immunosuppression methods to determine the minimum number of wt (Δa*kuA*) conidia that resulted in 100% mortality (not shown). Mice were immunosuppressed with CA (2 mg subcutaneously) administered on days −4, −2, 0 +2 and +4 in relation to infection, anesthetized with ketamine and xylazine and infected intratracheally with a target inoculum of 10^6^ conidia for wt or Δ*hacA* (n = 13) in PBS with 0.05% Tween 20. Five sham-infected mice were immune suppressed and then inoculated intratracheally with PBS containing 0.05 % Tween 20. Based upon plating efficiencies, mice received 1.1×10^6^ of the wt or 1.2×10^6^ conidia of the *ΔhacA* strains. Statistical significance was assessed by the log rank test using Sigma Stat 3.5

For the neutropenic model, mice were immunosuppressed with CPS (150 mg/kg intraperitoneally) and monoclonal antibody RB6-8C5 (25 µg intraperitoneally) one day before infection. CPS was then readministered three days after infection. Mice were infected intratracheally with a target inoculum of 5×10^5^ conidia of wt or Δ*hacA* (n = 13 or 12 per group, respectively) in PBS with 0.05% Tween 20. Five sham-infected mice were immunosuppressed and then inoculated intratracheally with PBS containing 0.05 % Tween 20. Based upon plating efficiencies using the infecting inoculum, mice received 4.3×10^5^ of the wt or 5.2×10^5^ conidia of the Δ*hacA* strains. Statistical significance was assessed by the log rank test using Sigma Stat 3.5.

For the triamcinolone (TA) immunosuppression model, conidia were harvested from plates of AMM supplemented with 1.2 M sorbitol and resuspended in sterile saline. Groups of 10 CF-1 outbred female mice (20–28 g) were immunosuppressed with a single dose of TA (40 mg kg^−1^ of body weight injected subcutaneously) on day −1. The mice were anaesthetized with 3.5% isofluorane and inoculated intranasally with 2×10^6^ conidia from the wt, *ΔhacA* mutant, or the complemented strain on day 0 in a 20 µl suspension. Mortality was monitored for 15 days, and statistical significance was assessed by ANOVA using Sigma Stat 3.5.

## Supporting Information

Figure S1 The Δ*hacA* mutant is hypersensitive to oxidative stress. 1×10^6^ conidia were spread evenly onto the surface of a 100 mm plate of IMA and a filter paper disk containing 15 µl of a 100 mg/ml solution of the superoxide-generating agent paraquat (methyl viologen) was placed onto the center. The plates were then incubated for 24 h at 37°C, and the zone of inhibition around the disk was used as an estimate of paraquat sensitivity.
(2.72 MB TIF)

Figure S2 Growth rate of Δ*hacA* at 42°C. A 5 µl suspension of conidia was spotted onto the center of an IMA plate and colony diameter was monitored for 3 days at 42°C. Values represent the mean of triplicate plates±SD. Colony morphology is shown after 3 days 42°C.
(0.81 MB TIF)

Figure S3 Hypersensitivity of Δ*hacA* to Congo red. A 5 µl suspension of conidia was spotted into the center of each well in a multi-well plate containing IMA and the indicated concentrations of Congo red and incubated for 3 days at 37°C. The experiment was repeated with similar results.
(5.62 MB TIF)

Figure S4 The experiment was performed as described in Fig. 8, except that IMA medium was used instead of RPMI agar, and a ketoconazole strip was used instead of a fluconazole strip. Amphotericin B (AmB), caspofungin (CS), itraconazole (IT), and ketoconazole (KE).
(1.79 MB TIF)

Figure S5 Antifungal susceptibility of *S. cerevisiae* Δ*hac1*. Yeast cultures were prepared as described in materials and methods prior to spread-plating onto YPD and overlaying with Etest strips containing caspofungin (A), ketoconazole (B), amphotericin B (C) or fluconazole (D). The plates were incubated for 48 h at 30°C.
(9.30 MB TIF)
