# Supplementary figures and images for: A Role for the Unfolded Protein Response (UPR) in Virulence and Antifungal Susceptibility in Aspergillus fumigatus
Source: PLoS Pathog. 2009 Jan 9;5(1):e1000258. doi: 10.1371/journal.ppat.1000258 (PMC2606855; doi:10.1371/journal.ppat.1000258)

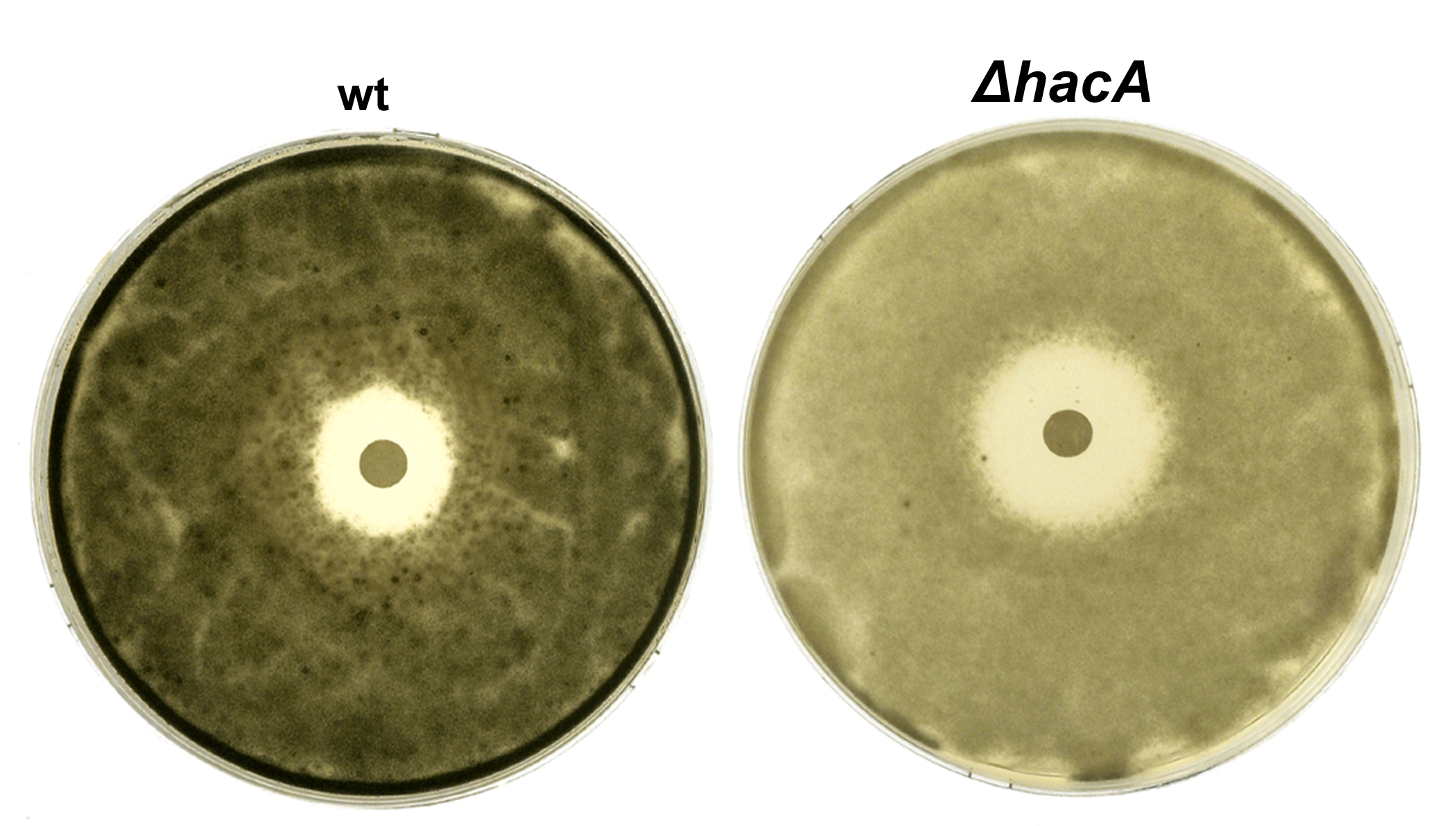

Supplement: Figure S1 — The ΔhacA mutant is hypersensitive to oxidative stress. 1×10^6^ conidia were spread evenly onto the surface of a 100 mm plate of IMA and a filter paper disk containing 15 µl of a 100 mg/ml solution of the superoxide-generating agent paraquat (methyl viologen) was placed onto the center. The plates were then incubated for 24 h at 37°C, and the zone of inhibition around the disk was used as an estimate of paraquat sensitivity. (2.72 MB TIF) [file ppat.1000258.s001.tif]

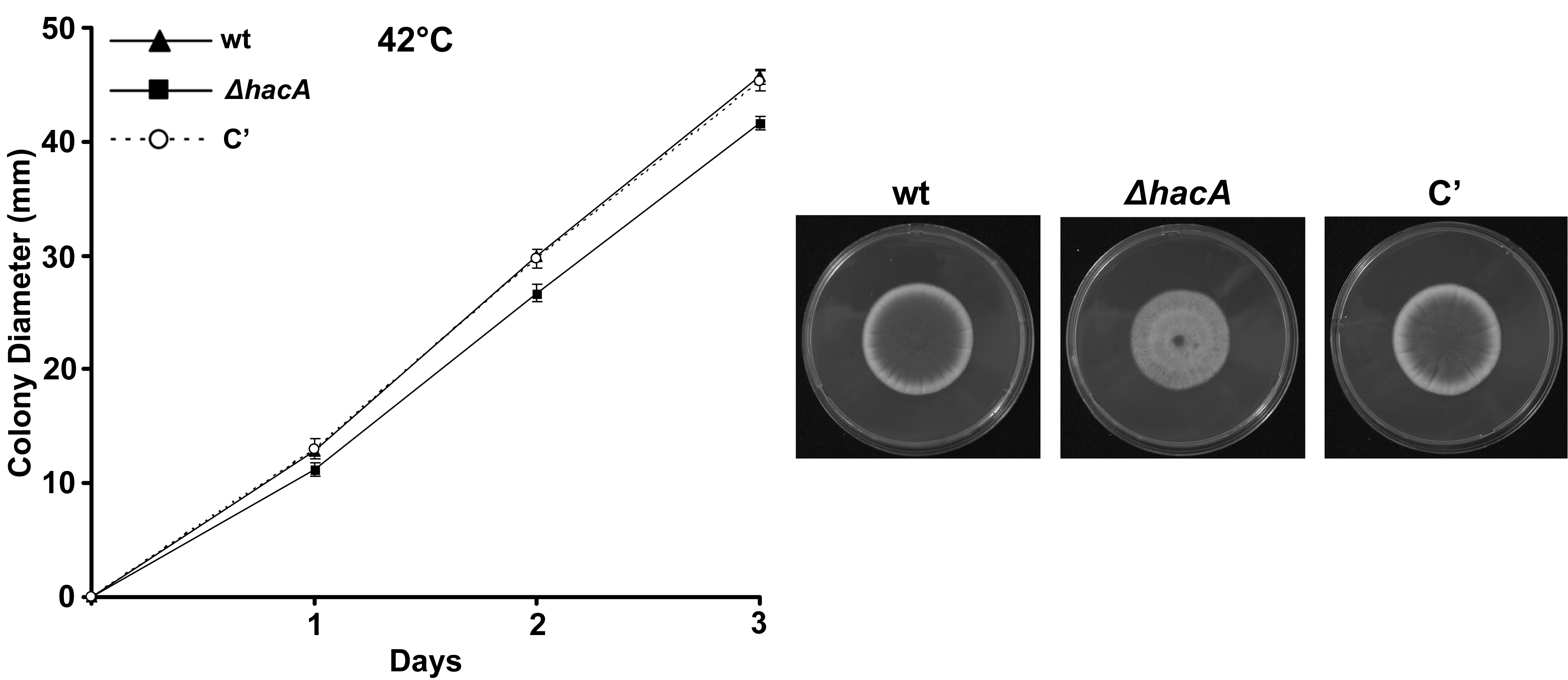

Supplement: Figure S2 — Growth rate of ΔhacA at 42°C. A 5 µl suspension of conidia was spotted onto the center of an IMA plate and colony diameter was monitored for 3 days at 42°C. Values represent the mean of triplicate plates±SD. Colony morphology is shown after 3 days 42°C. (0.81 MB TIF) [file ppat.1000258.s002.tif]

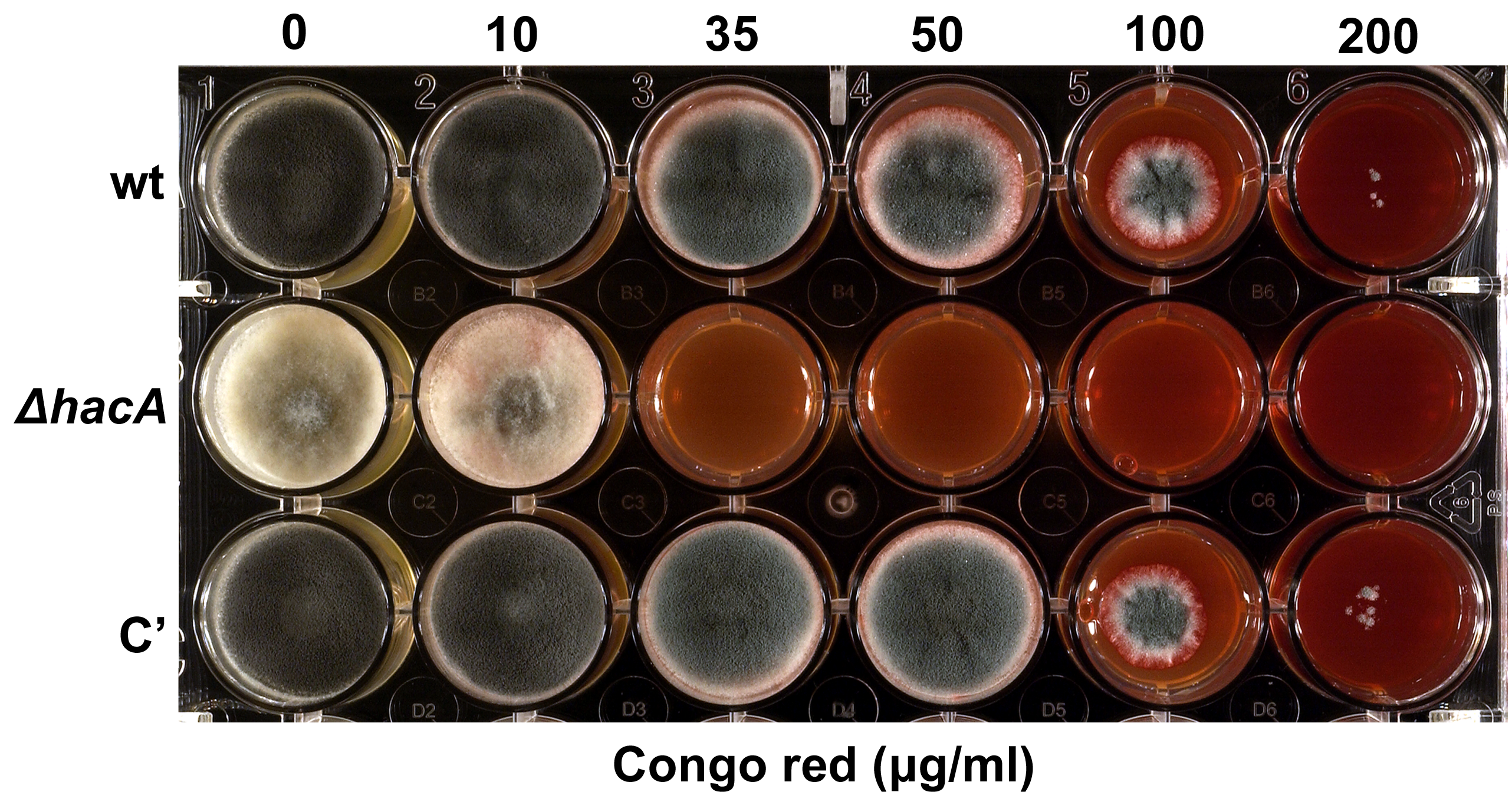

Supplement: Figure S3 — Hypersensitivity of ΔhacA to Congo red. A 5 µl suspension of conidia was spotted into the center of each well in a multi-well plate containing IMA and the indicated concentrations of Congo red and incubated for 3 days at 37°C. The experiment was repeated with similar results. (5.62 MB TIF) [file ppat.1000258.s003.tif]

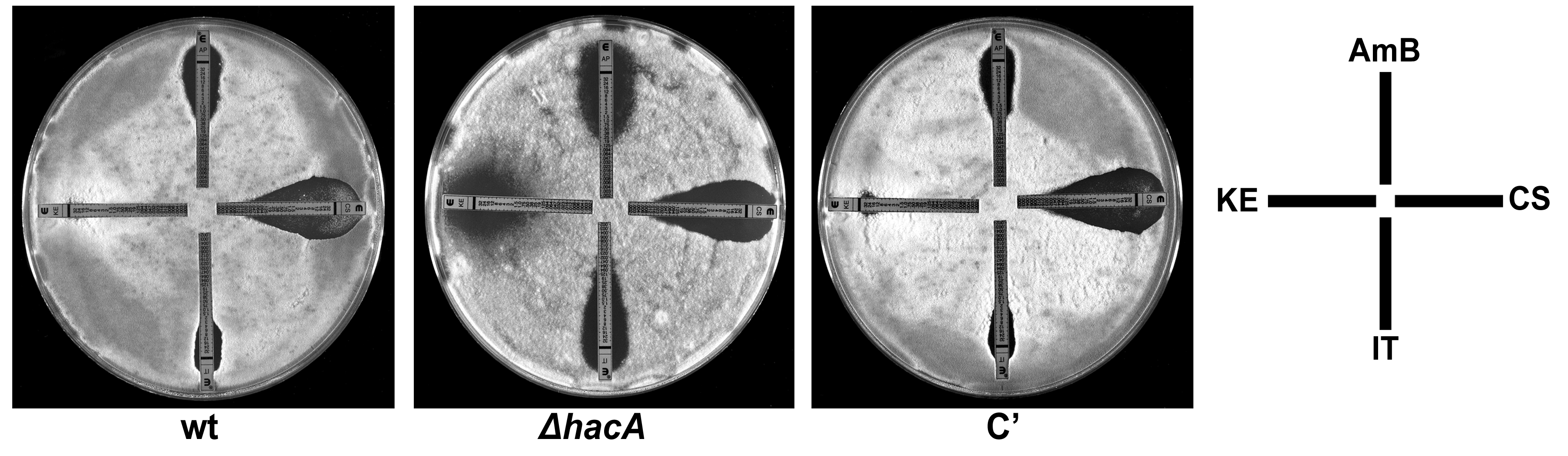

Supplement: Figure S4 — The experiment was performed as described in Fig. 8, except that IMA medium was used instead of RPMI agar, and a ketoconazole strip was used instead of a fluconazole strip. Amphotericin B (AmB), caspofungin (CS), itraconazole (IT), and ketoconazole (KE). (1.79 MB TIF) [file ppat.1000258.s004.tif]

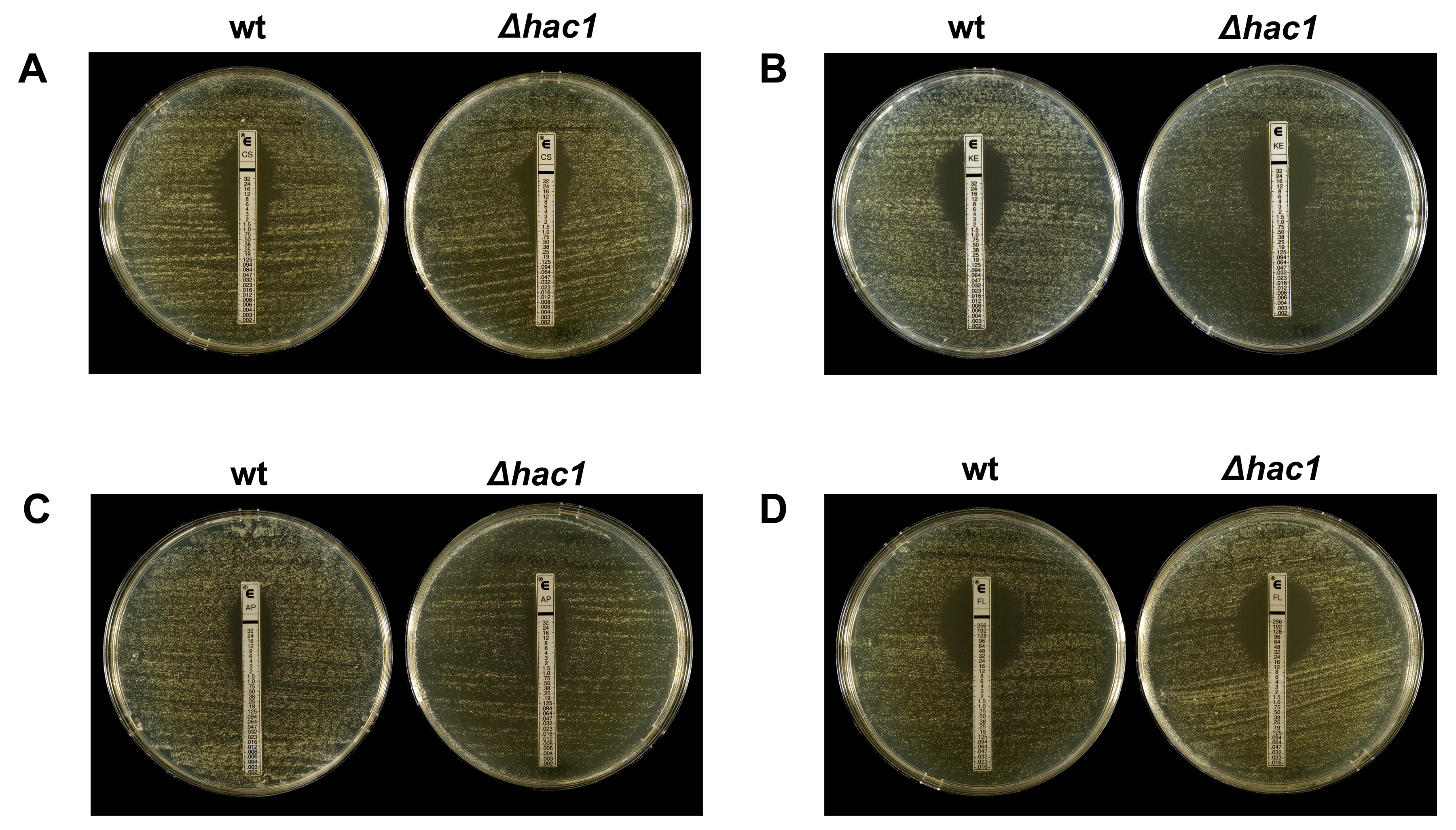

Supplement: Figure S5 — Antifungal susceptibility of S. cerevisiae Δhac1. Yeast cultures were prepared as described in materials and methods prior to spread-plating onto YPD and overlaying with Etest strips containing caspofungin (A), ketoconazole (B), amphotericin B (C) or fluconazole (D). The plates were incubated for 48 h at 30°C. (9.30 MB TIF) [file ppat.1000258.s005.tif]
